# Peripheral and Spinal Mechanisms Involved in Electro-Acupuncture Therapy for Visceral Hypersensitivity

**DOI:** 10.3389/fnins.2021.696843

**Published:** 2021-09-28

**Authors:** Adnan Hassan Tahir, Jia-Jia Li, Yong Tang

**Affiliations:** ^1^School of Acupuncture and Tuina and International Collaborative Centre on Big Science Plan for Purinergic Signalling, Chengdu University of Traditional Chinese Medicine, Chengdu, China; ^2^Acupuncture and Chronobiology Key Laboratory of Sichuan Province, Chengdu, China

**Keywords:** visceral hypersensitivity, electro-acupuncture analgesia, peripheral mechanism, spinal cord, neurological chemicals

## Abstract

One of the important characteristic features of clinically significant gastrointestinal disorders is visceral hypersensitivity (VH). Pain sensitization or VH is a big challenge for clinicians and becomes a very thorny work in clinical practices; the therapeutic efficacy for VH results in limited success. A popular second therapy that is being approved for the induction of analgesia and attenuates VH with fewer side effects includes electro-acupuncture (EA). Different peripheral and spinal neurological chemicals, including neurotransmitters, neuropeptides, and cytokines, and different signaling pathways were associated with EA treatment in VH. Despite the higher acceptance of EA, the underlying mechanism still needs to be further explored. In this paper, we review the available literature to find the peripheral and spinal mechanisms involved in EA to relieve VH.

## Introduction

The definition of visceral hypersensitivity (VH) includes hypersensitive visceral pain perception to colorectal distension (CRD), which is an experience by humans and animals having functional gastrointestinal (GI) disorders, such as inflammatory bowel disease (IBD) and irritable bowel syndrome (IBS) (Shah et al., 2016; Botschuijver et al., 2017). The mechanism involved in the pathogenesis of VH in IBD includes the spontaneous release of inflammatory mediators predominantly under the progression of an inflammatory attack and the recurrence that in turn causes sensitization of peripheral nerves in the enteric wall (Minderhoud et al., 2004; Bielefeldt et al., 2009). One of the most influencing factors causing abdominal pain in IBS is VH that is the contributing cause of triggering pain sensation in the bowel (Barbara et al., 2011). VH is a biological marker for IBS. The occurrence of VH in such long-term intestinal disorders has been known to cause and increase mortality rate and irreparable loss of the quality of life of humans and animals, resulting in a deem loss of economy on the worldwide basis (Zielińska et al., 2019). Hence, the underlying contributing factors include specific inflammatory processes, environmental, psychological, genetic, and microbiological parameters responsible for causing VH in IBD and IBS, but the exact mechanism of the cause of VH in these particular disorders is still unknown.

The clinical help seeking patients having IBS or IBD hold the consistent progression of VH. The pharmacological approach for the treatment of VH includes analgesics (non-steroidal anti-inflammatory drugs), antispasmodics, and antidepressants. Still, the use of such medications has been known to cause certain side effects that include insufficient pain relief, tolerance, and GI and cardiovascular toxicities (Davis, 2012). Some alternative therapies, including acupuncture, have been known to be effective treatment protocols that possess efficient therapeutic effects and minimum side effects compared with pharmacotherapy. As far as acupuncture is concerned, it can be defined as an oldest Chinese healing therapy that has been extensively used treatment protocol for humans and animals for over the last 3,000 years. Now, the modern Western medicine has also been convinced with the similarities as shown by the Chinese medicine (Bivins, 2001). In the present world, acupuncture therapy has been demonstrated to be a significantly popular and effective therapeutic for various diseases showing resistance to the conventional modes of treatment (Xie and Ortiz-Umpierre, 2006; Hutchinson et al., 2012).

**FIGURE 1:**
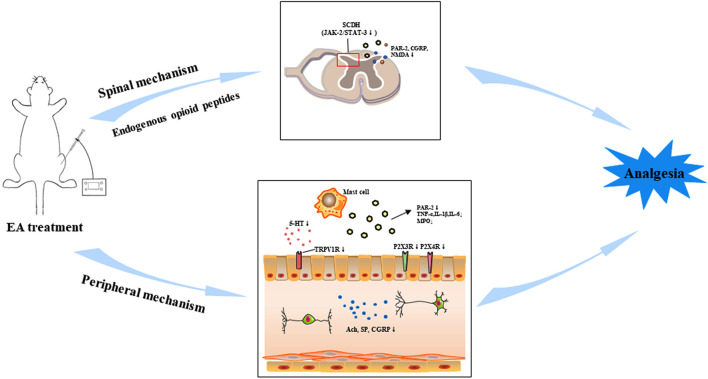
Electroacupuncture (EA) can relieve visceral hypersensitivity (VH) through peripheral and spinal pathways in the brain–gut axis by the regulation of different neurotransmitters and their receptors. Abbreviations: Acetylcholine (Ach), substance P (SP), 5-hydroxytryptamine (5-HT), transient receptor potential vanilloid 1 (TRPV1), tumor necrosis factor-alpha (TNF-α), interlukin-1β (IL-1β), interlukin-6 (IL-6), myeloperoxidase (MPO) enzyme, protease-activated receptor-2 (PAR-2), calcitonin gene-related peptide (CGRP), *N*-methyl-D-aspartate receptors (NMDAR), Janus kinase-2 (JAK-2)/signal transducer and activator transcription (STAT3).

Past studies have shown the relief of pain in response to acupuncture therapy as compared with the sham acupuncture or control groups. Acupuncture treatment is being associated with the change in the concentrations of β-endorphin, epinephrine, and serotonin and the levels of c-Fos, substance P, cytokines, P2X3, acetylcholine (Ach), *N*-methyl-D-aspartate (NMDA) receptors, and serotonin in the gut/spinal cord (Wan et al., 2017; Lee et al., 2019; Tan et al., 2019; Zhang et al., 2020). These studies show that electro-acupuncture (EA) can be used as an effective therapy to relieve VH. It is necessary to find more conclusive information to verify the effectiveness of EA and understand its basic mechanism. This review paper will summarize the effectiveness of EA to attenuate VH and try to find a new era of study in this field. Peripheral and spinal neurobiological mechanisms are involved in the attenuation of VH in different animal models by using acupuncture (Figure 1).

## Electro-Acupuncture Stimulus in Skin Could Regulate Nociceptive Processing in Intestines and Spinal Cord

EA is a modified form of traditional manual acupuncture (MA), in which it applies electrical stimulation to the peripheral nerves through the inserted needles at the acu-points. Most researchers use EA to prevent and treat different disorders because it can be easily standardized by voltage, frequency, and wavelength, it has better effects, and its stimulation can be objectively controlled and quantified. EA with different intensities can excite different types of peripheral afferent fibers and produce different extents of analgesia. The transmission of visceral nociception could be inhibited by EA in an intensity-dependent manner (Yu et al., 2019). EA analgesic effects are mediated through μ- and δ-opioid receptors at the lower frequency (2 Hz) and κ-opioid receptors at the higher frequency (100 Hz). At the two Hz frequencies, EA stimulates the release of β-endorphins, endomorphin, and encephalin. At 100 Hz, it releases dynorphin in the central nervous system (CNS) and binds to their corresponding receptors (Han, 2003, 2004). It is generally established that EA achieves analgesic effects on visceral pain *via* somatovisceral interactions on convergent neurons at different levels of the CNS (Wang et al., 2014). The wide dynamic range (WDR) neurons of the spinal dorsal horn could play an important role in managing visceral nociception (Zhang et al., 2011). These neurons receive a convergence of inputs from the external environment (the skin) and the internal milieu (the viscera, muscles, etc.) (Le Bars, 2002).

## Peripheral Mechanism of Electro-Acupuncture for Reducing Visceral Hypersensitivity

The present studies have shown that one of the promising conditions of visceral GI pain is VH. The peripheral and central mechanisms are involved in the pathogenesis of VH. The past studies have depicted that various harmful stimuli play a key role by affecting the receptors of the enteric mucosal linings resulting in the alertness of mast cells to secrete various inflammatory mediators, such as prostaglandins and bradykinin, thereby acting on the respective receptors of the sensory nerve endings (Mayer and Tillisch, 2011). Thus, nociceptive responses are transmitted to the spinal cord. The basic feature to study the neurological mechanism of EA for the relief of GI visceral pain has involved the study of neurons and the corresponding neurotransmitters and afferent fibers.

Acupuncture regulates the different peripheral neurobiological chemicals, including neurotransmitters, peptides, and cytokines, which cause to attenuate VH. In the intrinsic nervous system, cholinergic neurons of the intestinal myenteric plexus and sub-mucosal nerve plexus released an important neurotransmitter called Ach. Ach is considered as a significant neurotransmitter for regulating GI motility (McConalogue and Furness, 1994) and a powerful mediator of intestinal function (Yoo and Mazmanian, 2017), contributing to the primary afferents of acupuncture analgesic mechanism. Acupuncture may relieve visceral pain response by reducing the Ach level, which increased during inflammatory reactions (Yang et al., 2010).

The expression of substance P significantly lowered after using acupuncture (Liu et al., 2010; Yang et al., 2010). Substance P has also been known to play a key role in the progression of acupuncture analgesia (Karatay et al., 2018). EA at acupoint ST25 and ST37 significantly decreased VH response and mast cells count, substance P, neurokinin, certain vasoactive amines, and peptides in the colon of an IBS rat model induced with mechanical colorectal irritation (Wu et al., 2008; Xiao-Peng et al., 2009).

Another neurotransmitter serotonin [5-hydroxytryptamine (5-HT)] in the CNS and digestive tract regularizes various features of the digestive system. 5-HT and its receptors have been predominantly shown in the myenteric nerve plexus and participate in the different functions of the digestive tract (Huang et al., 2014). Previous research found an increase of 5-HT and its receptors in the intestine of patients suffering VH (Holtmann et al., 2016). 5-HT is involved in the regulation of activation of the transient receptor potential vanilloid 1 (TRPV1) (Winston et al., 2007). Thus, for the effective therapeutic treatment in VH-associated disorders, 5-HT is known to play a key role (Dunlop et al., 2005). EA stimulation decreased the colonic levels of 5-HT and 5-HT3 receptor expression in the brain–gut axis (Zhao et al., 2016). EA bilaterally at acupoint ST36 prominently lowered VH and colon 5-HT3 receptors expression in the IBS model induced by intrarectal acetic acid injection (Chu et al., 2011).

Different cytokines like tumor necrosis factor-alpha (TNF-α), interlukin-1β (IL-1β), and interlukin-6 (IL-6) and disease activity index, histopathological changes scour, and myeloperoxidase (MPO) enzyme expression increase in the inflamed intestine and have an important role in VH (Adam et al., 2013; Shah et al., 2016; Tahir et al., 2016). After acupuncture treatment in the 2,4,6-trinitrobenzene sulfonic acid (TNBS)-induced colitis or ileitis model, the level of TNF-α, IL-1β, and IL-6 and disease activity index, microscopic changes scour, and MPO level were decreased and caused to attenuate VH, and weight gain was improved (Tian et al., 2003; Kim et al., 2017; Shah et al., 2020).

A previous study showed that protease-activated receptor-2 (PAR-2) activation in different cells like granulocytes and certain agranulocytic white blood cells could induce the release of different inflammatory mediators, i.e., chemokines and prostaglandins, which promoted the production of neurogenic mediators, i.e., substance P and calcitonin gene-related peptide (CGRP), from the enteric nerve plexus and afferent nerves to ultimately trigger the VH (Cenac et al., 2003; Suckow and Caudle, 2009). Fecal supernatant of IBS patients was injected into the colon of healthy mice that resulted in higher mucosal inflammation and increased intestinal permeability, hence contributing to VH through a PAR-2 activation mechanism (Cenac et al., 2007; Annaházi et al., 2009; Shah et al., 2020). The respective findings have shown that activated PAR-2 in the intestine enhances VH. EA may relieve VH by reducing the mast cells number and the expression of PAR-2, substance P, CGRP, TRPV1, Toll-like receptor 4, and tryptase proteins in the colon tissue of IBS rats (Deng et al., 2018; Yang et al., 2019; Chen et al., 2021).

After the discovery of purinergic receptors by Burnstock, it is experimentally verified that adenosine triphosphate (ATP) is released from the epithelial cells of tubular organs or saclike organs after mechanical distension stimulation; this stimulation acts on the nerve plexus of the purinergic receptor P2X under the epithelial mucosa, thereby causing the transmission of pain signals to the pain center (Burnstock and Kennedy, 2011; Burnstock, 2014a). This assertion has been demonstrated in many GI disorders, such as IBS, IBD, and interstitial cystitis (Burnstock, 2014b). P2X receptors have been widely distributed in the body and are known to be involved in the formation, transduction, and regulation of neuropathic pain, inflammatory pain, and visceral pain or VH. Burnstock suggested that acupuncture mainly acts through purinergic signals (Burnstock, 2011). Purinergic signaling has recently been suggested to constitute the cellular mechanism underlying acupuncture-induced analgesia. By extending the original hypothesis on endogenous opioids being released during acupuncture analgesia, Burnstock and Nedergaard provided evidence for the involvement of purinergic receptors (P2 and P1/A1 receptors) in the beneficial effects of acupuncture-induced analgesia (Tang et al., 2018). This statement provided new ideas for the study of the mechanism involving acupuncture and depicted new challenges (Huang et al., 2021).

A previous study demonstrated that EA could regulate the expression of the P2X3 receptors in the peripheral and central pathways of visceral pain transmission to achieve remission of VH in the IBS rat model (Weng et al., 2015). EA downregulated the increased expression of P2X3 receptors in the colonic myenteric plexus and colon-associated dorsal root ganglion (DRG) neurons and is involved in the attenuation of VH in rats with the IBS model (Zhang et al., 2020). EA can attenuate VH in the IBS rat model by decreasing the expression of P2X4 receptors in the colon (Guo et al., 2013). There is further need to explore the mechanism of EA on the brain–gut neural signal transmission at different levels, including the enteric nervous system in VH, and investigate the other P2X and P2Y receptors. Therefore, it can be said that EA can downregulate different peripheral chemicals to lower the sensitization process of the enteric nerves and have the effect to alleviate VH (Figure 1). Despite that fact, there is a vital need to investigate the new experimental designs that concern the specific agonists, antagonists, and gene knockouts to find more convincing information to understand the peripheral neurobiological mechanism of EA involved in the attenuation of VH.

## Spinal Mechanism of Electro-Acupuncture for Reducing Visceral Hypersensitivity

There is a strong covenant that VH is associated with spinal cord sensitization, which involves different excitatory neurotransmitters (Bueno et al., 1997; Khan et al., 2006; Kapural et al., 2010; Baranidharan et al., 2014). In the spinal cord dorsal horn (SCDH), different vital afferent nerves converge from the periphery, SCDH neurons, and descending nerves from the superior center, thereby forming a neuron network having complex characteristics. The spinal cord is composed of various neurotransmitters and the corresponding receptors, certain neuromodulators, and various ion channels that are involved in the reception and transmission of nociceptive information (Luo et al., 2014; Zhang et al., 2017). Inhibition of central sensitization causes to alleviate visceral pain or VH (Sarkar et al., 2001).

Acupuncture stimulation may inhibit the neural response activated by enteric nociceptive afferents and be responsible for the induction of the attenuation of visceral pain or VH (Yu et al., 2014). A previous study found that neuromodulators, especially some endogenous opioid peptides (endorphin, enkephalin, and dynorphin), have significant contributions in EA analgesia (Przewłocki et al., 1986). EA can boost the release of different opioids, which act on their related receptors to exert an analgesic effect. EA can inhibit the effect of different neurotransmitters involved in pain sensitization by regulating endogenous opioids in the DRG and spinal cord, resulting in the attenuation of visceral pain response (Qi et al., 2016).

c-Fos has been exclusively considered a biological indicator to examine the neuronal activity, as the expression of c-Fos boost in the spinal cord causes the excitement of the CNS. The continuous or subsequent nociceptive stimuli increase spinal cord c-Fos expression, contributing to the generation of a pain state partly because of adaptive spinal response (Qiu et al., 2015). Therefore, c-Fos is providing the active site for evaluating the active substances or treatment in the CNS. Researchers have also established that EA reduced peripheral inflammation and c-Fos expression in the spinal cord (Xu et al., 2010; Qi and Li, 2012; Shah et al., 2020). The c-Fos expression level increased in the SCDH superficial lamina during VH with TNBS-induced ileitis but decreased after EA therapy (Shah et al., 2020). This phenomenon indicating that ileitis-induced VH activated the SCDH superficial lamina was the important site on which EA regulates its effects. This indication shows that EA inhibits the transmission of noxious stimulation at the spinal cord level, which may involve the participation of different neurobiological molecules. EA reduces the expression of c-Fos, p38, and 5-HT receptors and ultimately causes the attenuation of pain sensitization (Wu et al., 2010; Xu et al., 2010). EA can reduce the level of c-Fos and different NMDA excitatory receptors in the spinal cord and attenuate VH (Tian et al., 2008; Wang et al., 2009).

The agonist of PAR-2 enhances the potassium chloride ion and CGRP in the sensory neuron (Steinhoff et al., 2000; Hoogerwerf et al., 2001). The role of CGRP in VH has been confirmed by using CGRP gene knockout and antagonist of CGRP receptor in rodents (Delafoy et al., 2006; Thompson et al., 2008). EA can attenuate VH by reducing the expression of c-Fos, PAR-2, and CGRP in the SCDH in the rats with IBD and IBS models (Sun et al., 2015; Shah et al., 2020).

The Janus kinase (JAK)/signal transducer and activator transcription (STAT) pathway activated by cytokines is the key signaling mechanism in central sensitization. JAK-2 specifically activates the STAT3 in the spinal cord and involves VH (Sriram et al., 2004; Bradesi et al., 2015). IL-6 is one of the important up-streaming pro-inflammatory cytokines of the JAK-2/STAT3 pathway. In goats with TNBS-induced ileitis, TNBS enhanced the visceral motor response (VMR) and pain behavior response to CRD and increased the expression of IL-6, JAK-2, and STAT3 in the SCDH, periaqueductal gray (PAG), and rostral ventromedial medulla (RVM). EA treatment attenuated the VMR and pain behavior response and decreased the IL-6, JAK-2, and STAT3 expression in the SCDH, PAG, and RVM (Wan et al., 2017). EA relieved the VH primarily by inhibiting the JAK-2/STAT3 signaling pathway in the PAG–RVM–SCDH axis (Wan et al., 2017).

It is known that for approximately 50 years, the nucleotides are known to perform a critical role in the induction of nociception and have an essential role in the mechanisms of pain (Burnstock, 2016). Extracellular ATP induces its downregulation signaling through activation of two major P2 purinergic receptors. P2X is the ionotropic, and P2Y is the metabotropic G protein-coupled receptors. As we discussed previously, the purinergic receptors in the peripheral and CNS are involved in the process of visceral hypersensitization in IBS. Acupuncture may attenuate the VH by regulating the purinergic receptors. EA can attenuate VH by reducing purinergic P2X3 receptor expression in the intestine and CNS by regulating brain–gut neural signal transmission (Weng et al., 2015). Still, there is a need to find a more detailed neural mechanism by which EA is involved in the attenuation of VH in the spinal cord under purinergic signaling molecules.

## Conclusion

EA mechanisms involved in the attenuation of VH have been extensively studied. This review demonstrated the attenuation of VH following EA treatment. The EA treatment causes inhibition of both the sensory and respective affective components of VH *via* the routes of the peripheral and spinal cord, by the involvement of aggregation of bioactive neurobiological molecules including opioids, cytokines (TNF-α, IL-1β, and IL-6), serotonin, substance P, NK1, PAR-2, CGRP, glutamatergic NMDA receptors, purinergic P2X3 receptor, P38, JAK-2, and STAT3 signaling molecules (Figure 1). How these neurobiological molecules and structures (SCDH and visceral organs) combined work together remains unidentifiable. Purinergic signaling molecules are more recent players in the field of EA analgesia (Tang et al., 2018; He et al., 2020), but only few studies are available about the analgesia effects of EA on visceral organs. Still, there is a need to find a more detailed neural mechanism by which EA is involved in the attenuation of VH in the intestine and spinal cord under the purinergic signaling pathway. There is also a need to investigate the new experimental designs that concern the specific agonists, antagonists, and gene knockouts to find more convincing information to understand the peripheral and spinal neurobiological mechanisms of EA involved in the attenuation of VH. Additionally, the intestinal microbiome is recognized to be responsible for the control of VH (Li et al., 2020; West and Neufeld, 2021). It is very necessary to punch the truth whether the intestinal microbiome or gut–brain–microbiota axis (Yaklai et al., 2021) plays a crucial role in acupuncture reducing VH. Another recent report indicated that inhibiting the activation of astrocytes in the medial thalamus and anterior cingulate cortex would get involved in EA alleviating IBS VH. It implied that the central mechanism should not be neglected in the future research (Zhao et al., 2020).

## Author Contributions

AT and J-JL drafted the manuscript. All authors discussed, edited, and approved the final version for submission.

## Conflict of Interest

The authors declare that the research was conducted in the absence of any commercial or financial relationships that could be construed as a potential conflict of interest.

## Publisher’s Note

All claims expressed in this article are solely those of the authors and do not necessarily represent those of their affiliated organizations, or those of the publisher, the editors and the reviewers. Any product that may be evaluated in this article, or claim that may be made by its manufacturer, is not guaranteed or endorsed by the publisher.
